# Dipeptidyl Peptidase-4 at the Interface Between Inflammation and Metabolism

**DOI:** 10.1177/1179551420912972

**Published:** 2020-03-20

**Authors:** Natasha A Trzaskalski, Evgenia Fadzeyeva, Erin E Mulvihill

**Affiliations:** 1 University of Ottawa Heart Institute, Ottawa, ON, Canada; 2 Department of Biochemistry, Microbiology and Immunology, Faculty of Medicine, University of Ottawa, Ottawa, ON, Canada

**Keywords:** Dipeptidyl peptidase-4, incretin hormones, inflammation, glycemia, insulin resistance, type 2 diabetes, nonalcoholic fatty liver disease

## Abstract

Dipeptidyl peptidase-4 (DPP4) is a serine protease that rapidly inactivates the incretin peptides, glucagon-like peptide-1, and glucose-dependent insulinotropic polypeptide to modulate postprandial islet hormone secretion and glycemia. Dipeptidyl peptidase-4 also has nonglycemic effects by controlling the progression of inflammation, which may be mediated more through direct protein-protein interactions than catalytic activity in the context of nonalcoholic fatty liver disease (NAFLD), obesity, and type 2 diabetes (T2D). Failure to resolve inflammation resulting in chronic subclinical activation of the immune system may influence the development of metabolic dysregulation. Thus, through both its cleavage and regulation of the bioactivity of peptide hormones and its influence on inflammation, DPP4 exhibits a diverse array of effects that can influence the progression of metabolic disease. Here, we highlight our current understanding of the complex biology of DPP4 at the intersection of inflammation, obesity, T2D, and NAFLD. We compare and review new mechanisms identified in basic laboratory and clinical studies, which may have therapeutic application and relevance to the pathogenesis of obesity and T2D.

## Introduction

Dipeptidyl peptidase-4 (DPP4) is a protease with a well-characterized role in regulating the bioactivity of gastrointestinal-derived peptide hormones, leading to significant implications for endocrine pathways.^1,2^ Inhibition of DPP4-mediated degradation of gut hormones potentiates islet hormone secretion and enhances postprandial metabolism to successfully treat hyperglycemia in patients with type 2 diabetes (T2D).^3^ Dipeptidyl peptidase-4 has also cleaves and inactivates several chemokines and cytokines with a significant impact on inflammation and immune function.^4^ Dipeptidyl peptidase-4 can also directly cleave the extracellular matrix and influence cell migration.^5,6^

**Figure 1.**
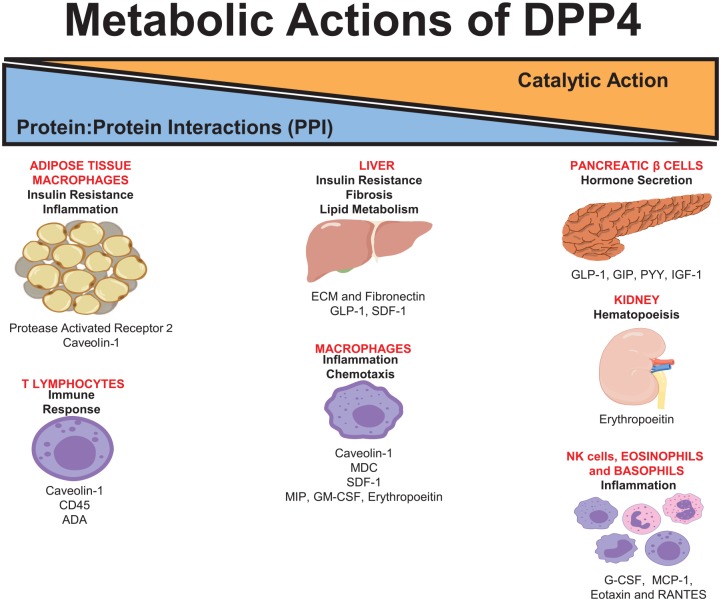
Schematic illustrating the metabolic consequences and cellular specificity of dipeptidyl peptidase-4 regarding glucose tolerance and inflammation and the evidence indicated to date on substrate cleavage and regulation vs noncatalytic/direct protein-protein interactions as the mechanisms underlying these effects. ADA indicates adenosine deaminase; G-CSF, granulocyte colony-stimulating factor; GIP, glucose-dependent insulinotropic polypeptide; GLP-1, glucagon-like peptide 1; GM-CSF, granulocyte macrophage colony-stimulating factor; IGF-1, insulin growth factor; MDC, macrophage-derived cytokine; MIP-1, macrophage inflammatory protein 1; PYY, peptide tyrosine tyrosine; SDF-1, stromal-derived factor 1.

Interestingly, both activation of intracellular signaling cascades regulating immune cell activation and its interaction within a complex to influence extracellular matrix proteolysis can occur independently of catalytic activity.^5,7^ Therefore, dissection of the actions mediated by the catalytic activity and posttranslational regulation of its host of substrates versus those mediated by cleavage-independent or direct protein interactions with cell surface receptors, extracellular matrix, and cell signaling cascades is currently important given its emerging role as a biomarker of metabolic disease.^8^ Recent research has placed these distinct functions at a direct intersection, where regulation of inflammation may influence the progression of dysglycemia, insulin resistance, obesity, and nonalcoholic fatty liver disease (NAFLD) (Figure 1). Here, we discuss and contrast the potential substrates identified with those experimental observations which appear to be independent of catalytic activity and highlight relevance for human disease.

## DPP4 Structure and Catalytic Activity

Dipeptidyl peptidase-4 is a type II cell surface exopeptidase with a classic serine triad defining the C-terminal catalytic active site and a single hydrophobic sequence anchoring the mostly extracellular protein (only 6 amino acids extend into the cytoplasm in the membrane).^9^ Dipeptidyl peptidase-4 belongs to the prolyl endopeptidase family, a group of atypical serine proteases with an active site consisting of the catalytic residues Ser630, Asp708, and His740, which hydrolyze a prolyl bond in substrate proteins.^2,9-13^

## Transcriptional Control of DPP4

Dipeptidyl peptidase-4 contains a GC-rich sequence.^14,15^ This region contains several consensus binding sites for transcriptional factors, including hepatocyte nuclear factor-1-beta (HNF1B),^16^ cut-like homeobox 1 (CUX1)^17^ glucocorticoid receptor,^6^ and specificity protein 1 (SP1),^18^ as well as several cytokines, such as interferon-γ and tumor necrosis factor α (TNF-α), which regulate *Dpp4* messenger RNA (mRNA) expression in a cell type–specific manner.^18^ Promoter analysis has also identified consensus sites for nuclear factor kappa-light-chain-enhancer of activated B cells (NF-κB) and epidermal growth factor activating protein 1.^19^ In HepG2 cells, incubation with high concentrations of glucose increases the expression of *Dpp4*^20^ and has been confirmed by luciferase assays.^21,22^ Positive correlations have been established with plasma DPP4 activity and fasting plasma glucose^23^and HbA1c^24^ However, this regulation may be more complicated in vivo as patients with improved glycemic control do not consistently experience a reduction in circulating DPP4,^25^ and short-term treatment of mice with the glucose-lowering agent Exendin-4 does not reduce circulating DPP4 activity.^26^ Incubation of HepG2 cells with insulin, palmitate, oleate, or cholesterol did not result in increased *DPP4* mRNA expression. In THP-1 macrophages, dexamethasone treatment significantly induced transcriptional upregulation of *DPP4* due to the presence of two glucocorticoid responsive elements within the promoter.^6^

## Posttranscriptional Regulation of DPP4

Dipeptidyl peptidase-4 exerts enzymatic activity in both the membrane-anchored and circulating soluble form,^2,9,12^ and it requires heterodimerization or homodimerization for catalytic function.^13^ As a dimer, DPP4 selectively and preferentially cleaves a dipeptide from the N-terminus with a position 2 proline or alanine and a protonated amino terminus.^11^ Dipeptidyl peptidase-4 can be posttranslationally modified, including glycosylation (sialylation) at several sites responsible for targeting DPP4 to the apical membrane.^27^ Particularly noteworthy is the N-glycosylation at the Asn 319 site, which when mutated significantly reduces dimerization and catalytic activity.^28^ In addition, DPP4 can also be oxidized, which reduces its activity.^29^

Dipeptidyl peptidase-4 is present on the membrane of parenchymal cells within metabolic organs, including hepatocytes, enterocytes, islets cell, and within endothelial cells and immune populations.^26,30^ The level of expression depends on the cell type, differentiation state, and/or the activation state.^4,31,32^ Dipeptidyl peptidase-4 can also be shed from the membrane and circulates throughout many bodily fluids.^33^ Sheddases are a class of membrane-bound enzymes that can cleave transmembrane proteins and have been proposed for the release of DPP4 from the cell membrane into circulation as classic endothelial reticulum/Golgi secretion pathways are not involved.^34^ Matrix metalloproteinases (MMP) 1, 2, 14, and 9 and Kallikrein-related peptidase 5 (KLK5) have all been identified to have a role in shedding.^33^ However, the contribution, regulation, and cell-type specificity of these sheddases to the regulation of soluble DPP4 and disease progression are currently unknown.

## Direct Protein-Protein Interactions With DPP4

In addition to its well-described peptidase activity, DPP4 also possesses noncatalytic functions through its interaction with ligands, including adenosine deaminase (ADA), caveolin-1,^35^ extracellular matrix (collagen and fibronectin), and C-X-C chemokine receptor 4 (CXCR4).^19^ Dipeptidyl peptidase-4 is a co-stimulator for T-cell activation by interaction and activation of ADA. As adenosine is a potent suppressor of T-cell proliferation, inducing its degradation through increased ADA activity induces T-cell proliferation. However, studies using DPP4 with a mutation within the active site rendering it catalytically inactive or a mutant DPP4 unable to bind ADA, demonstrated that DPP4 induces T-cell proliferation through pathways independent of ADA and substrate degradation.^36^ Dipeptidyl peptidase-4 has also been proposed to bind directly to CD45 to induce T-cell receptor signaling.^37^ However, given that mice with genetic elimination of *Dpp4* or treatment with a highly selective DPP4 inhibitor (DPP4i) had comparable and robust primary and secondary antibody responses to T-dependent antigens provides compelling evidence that although DPP4 has a role in mediating T-cell activation, it is not absolutely required for T-cell–directed immune responses.^38^ Evidence also exists that DPP4 physically interacts with caveolin-1 on antigen-presenting cells to induce aggregation and phosphorylation, which activates NF-κB.^39^ In addition, DPP4 has been demonstrated to activate signaling on endothelial cells through direct interaction with the mannose 6 phosphate/insulin-like growth factor 2 receptor.^40,41^

### DPP4 and regulation of the bioactivity of incretin hormones

The best-characterized substrates regulated by DPP4 catalytic activity are glucagon-like peptide-1 (GLP-1) and glucose-dependent insulinotropic polypeptide (GIP), which are responsible for the incretin effect or 60% of insulin secreted in response to nutrients.^42^ Both the GLP-1 receptor (GLP-1R) and GIP receptor (GIPR) belong to the G-protein–coupled receptor B1 superfamily, which activates G_αs_ proteins and stimulates cyclic adenosine monophosphate (cAMP) production upon ligand binding.^43^ Both GLP-1 and GIP in circulation are degraded by DPP4 cleavage of the N-terminal two amino acids very efficiently, eliminating their glucoregulatory action due to a decrease in their respective receptor’s affinity.^1^ Meal-induced spikes in circulating concentrations sufficient to reach the islet last only minutes.^44^ Most degradation occurs within the vessels draining the mesentery and within the portal circulation and hepatic bed.^30,45-48^ However, the ablation of osteoclasts through treatment with denosumab has been reported to reduce circulating DPP4 levels and raise GLP-1, suggesting many metabolic circumstances may influence DPP4 levels and incretin cleavage.^49^ Inhibition of degradation of GLP-1 and GIP by pharmacological inhibitors preserves the bioactivity of active incretins, allowing direct activation of incretin receptors on the β cell to augment meal-stimulated insulin secretion. In addition, GLP-1 inhibits glucagon secretion through activation of GLP-1 receptors on δ cells to stimulate somatostatin secretion.^50^

### DPP4 inhibitors for the treatment of hyperglycemia in patients with T2D

Dipeptidyl peptidase-4 inhibitors (DPP4i) are approved for the treatment of hyperglycemia in patients with T2D. They are highly selective for and lead to significant inhibition of the catalytic activity of DPP4, ultimately functioning by preventing the degradation of the incretin hormones, stimulating postprandial insulin secretion,^2,8^ and reducing hepatic glucose production through lowered glucagon secretion.^51^ Generally, the structures of DPP4i fall within 3 broad categories: (1) a substrate-like electrophilic group that can interact either covalently or noncovalently with the active binding site of DPP4, (2) non–substrate-like inhibitors, or (3) xanthine-based compounds.^52^ DPP4i are relatively well tolerated as the risk of hypoglycemia is low, given the glucose dependence of incretin-mediated insulin release. Overall, DPP4i do not have any reported impact on body weight, blood pressure, or heart rate, and treatment results in small improvements in the aberrant lipid profile associated with T2D, including reduced triglycerides (TG) and increased high-density lipoprotein cholesterol.^53^ All the cardiovascular outcome trials of DPP4i have reported significantly improved glycemic control and met the safety requirements of a neutral effect on major adverse cardiovascular events,^54-57^ despite being powered to demonstrate the expected benefit on cardiovascular disease inferred from ample preclinical data (as reviewed elsewhere).^58-62^ In addition to glycemic control, DPP4i have also demonstrated to be effective erythropoiesis-stimulating agents for renal anemia through the preservation of active erythropoietin.^63,64^ Also, although B-type natriuretic or brain peptide is a substrate for DPP4 cleavage, inhibition of DPP4 with linagliptin did not affect N-terminal pro–brain natriuretic peptide (BNP) levels or BNP in patients with T2D.^65^ It has also demonstrated that sitagliptin treatment of healthy subjects does not potentiate vasodilation in response to BNP injection.^66^

## DPP4 Inhibitors in Combination With Metformin

DPP4i are often prescribed together or provided as a dual therapy when metformin is not sufficient to maintain euglycemia.^67-73^ Patients who receive dual therapy experience additive glucose-lowering and superior improvements in HbA1c.^74^ Metformin has been demonstrated to mediate significant metabolic effects through its action within the gastrointestinal tract.^75,76^ Preproglucagon (*Gcg*) is expressed in enteroendocrine L cells, which are dispersed throughout the duodenum but more concentrated toward the distal colon.^77,78^ Glucagon-like peptide-1 is processed from the preproglucagon polypeptide by prohormone convertase 1/3 (PC1/3), and its secretion is stimulated by ingested nutrients.^44,79,80^

One proposed gut-based mechanism of action for metformin is to stimulate GLP-1 secretion directly from the L cell.^81^ In addition, metformin may also enhance GLP-1R and GIPR expression within the β cell.^82,83^ Consistent with increased secretion, nondiabetic, obese patients who received metformin monotherapy experience an increase in active GLP-1, and metformin did not alter the kinetics of GLP-1 degradation by DPP4.^84,85^ Metformin is, therefore, an effective partner for additive therapy with a DPP4i. In addition to the superior lowering of HbA1c, dual therapy is a promising course of treatment for reducing adverse cardiovascular events. A recent post hoc subgroup analysis of the three cardiovascular outcome trials for DPP4i suggests that patients treated at baseline with metformin may derive cardiovascular benefit from DPP4i compared with metformin nonusers.^86,87^

### Role of endothelial cell–derived DPP4 in glycemic regulation

Both *Dpp4*^−/−^mice and F-344/DuCrj rats (deficient in DPP4 catalytic activity) demonstrate increased GLP-1 concentrations, higher insulin secretion, and improved postprandial glucose control.^88-90^ Activation of the GLP-1R present on β cells regulates not only hormone secretion from endocrine cells potentiating glucose-stimulated insulin secretion and reducing glucagon secretion through stimulation of somatostatin but also increases insulin synthesis, β-cell neogenesis, islet mass, and β-cell survival.^91-93^ Dipeptidyl peptidase-4 is present on endothelial cells throughout the body, including those adjacent to the enteroendocrine L cells which produce GLP-1.^94^ Consistent with these observations, enteric targeting of DPP4 by genetic elimination of *Dpp4* from endothelial cells or low pharmacological concentrations of DPP4 inhibitors can significantly increase circulating, active GLP-1 concentrations and improve glycemia.^8,30,95^ The portal circulation and hepatic bed have also been identified as DPP4+ sites for GLP-1 degradation.^30,45-48,96^ However, it is most likely endothelial cells within the portal system which regulate the cleavage as elimination of *Dpp4* specifically from hepatocytes or administration of a small interfering RNA (siRNA) to target *Dpp4* within hepatocytes failed to augment circulating incretin levels and improve glucose tolerance.^26,97^

The proGIP gene encodes the incretin hormone GIP, which is mainly expressed in the intestinal K cells.^44^ Similar to GLP-1, PC1/3 converts a proGIP prohormone precursor into active GIP. In addition to being an incretin hormone, GIP signaling and GIPR have been shown to play a role in diet-induced obesity,^98^ adipokine secretion,^99^ lipoprotein lipase activity, and TG accumulation.^100^ Degradation of significant amounts of GIP by DPP4 occurs within in the gut by both endothelial and immune cell populations.^30^

### Role of islet-derived DPP4 in glycemic regulation

Recent evidence in mice genetically engineered to reexpress *Gcg* has suggested that bioactive and glucoregulatory GLP-1 may be produced in the pancreas.^101^ Dipeptidyl peptidase-4 protein expression has been observed in isolated human islets,^102^ and GLP-1R antagonism blunts the DPP4i (linagliptin) improvement in mice with *Gcg* expression restricted to the pancreas,^103^ suggesting a potential pancreatic, paracrine circuit to control glycemia. However, further use of these models to reexpress *Gcg* in the proximal and distal gut demonstrates gut-derived GLP-1 is indeed the dominant site of GLP-1, and pancreas perfusion studies with a DPP4i have demonstrated no impact on glycemia.^104^ However, recently, *Dpp4* mRNA and DPP4 protein have been proposed to be expressed dynamically in late fetal stages of β-cell development, which coincide with the expression of GLP-1R and may regulate GLP-1–mediated signaling responses important for β-cell maturation.^105^ The importance of preproglucagon-derived peptides in the control of islet cell communication through cAMP has been recently demonstrated,^106,107^ and the addition of sitagliptin to metformin is associated with a lower rate of diabetes progression.^108^ These data suggest that although an islet-DPP4-GLP-1 axis may not regulate whole-body glucose metabolism, it may regulate necessary islet-specific signaling important in β-cell function and survival.

Both the mRNA of proGIP and a biologically active, truncated form of GIP (1-30) have been localized to alpha cells of both mouse and human islets, which is stimulated and secreted in response to arginine.^109^ The DPP4-mediated degradation product GIP 3-42 has also been demonstrated to act as a GIPR antagonist.^43^ However, the importance of DPP4-mediated regulation of GIP within the islet is complicated by observations that in states of T2D, the GIPR undergoes desensitization and degradation.^110-113^

Peptide tyrosine tyrosine (PYY) is another DPP4 substrate secreted by endocrine L cells and pancreatic α cells in response to glucose and is associated with insulin secretion.^114^ Cleavage of PYY(1-36) by DPP4 leads to the creation of truncated peptide, PYY(3-36), which exhibits altered receptor selectivity. Unlike PYY(1-36) that binds to all NPY receptor subtypes with equal affinity, the truncated PYY(3-36) exhibits high-affinity binding to the NPY2 receptor.^115^ PYY(1-36) was shown to inhibit glucose-stimulated insulin secretion in isolated islets, whereas PYY(3-36) had no effect.^116^ Also, administration of exogenous PYY(3-36) during an intraperitoneal glucose tolerance test has been shown to lower glycemia through GLP-1–mediated improvements in insulin secretion, whereas PYY(1-36) did not.^116-118^ Further studies using native PYY (1-36) have demonstrated β-cell proliferation and protection from cytokine-induced apoptosis in BRIN BD11 and 1.1B4 cells.^119^ As levels of intact and DPP4 cleavage products of PYY have been difficult to measure, the biological relevance of islet cleavage in the regulation of glucose is uncertain.

## DPP4 as a Regulator of Immune Cells

Consistent with the promoter analysis, altered expression of DPP4 in autoimmune and infectious diseases, hematological cancers, and tumors has been reviewed in detail elsewhere.^4,7,11,120^ There are several significant differences between mice and humans in this regard, as DPP4 expression in the hematopoietic compartment differs between them. In human subjects, DPP4 is expressed predominantly by T cells, whereas in mice, dendritic cells, B cells, and natural killer cells also express significant levels of DPP4.^121^ Lipopolysaccharide treatment of mice increases expression of DPP4 on macrophages 5- to 10-fold, suggesting acute activators of inflammation are associated with significantly increased DPP4 expression in circulating immune cells.^22^ Consistent with this, the release of DPP4 is induced by treatment with TNF-α and with insulin.^122^ The effects of low-grade, often subclinical inflammation observed in patients with metabolic disease offers less clarity in the form of DPP4 expression on immune cell populations. Assessment of 14 controls versus 27 patients with confirmed atherosclerotic plaque identified that DPP4 is elevated on CD11b+ monocytes, and it correlates with plasma TG and cholesterol, but not with glucose or insulin concentrations.^123^

Peripheral blood mononuclear cells isolated from patients with T2D demonstrated no difference in DPP4 activity or gene expression.^124^ It has been reproducibly demonstrated that a significant portion of circulating DPP4 originates from bone marrow–derived cells,^30,121,125,126^ suggesting they are an essential reservoir of soluble DPP4. In patients with severe combined immunodeficiency, as well as studies in irradiated mice, the number of lymphocytes in circulation correlated with levels of circulating DPP4.^121^ Additional evidence suggests DPP4 can derive from osteoclasts and signal through receptor activator of nuclear factor-kappa B ligand (RANKL) under circumstances of bone remodeling.^49^ RNA-seq data have identified DPP4 as one of the factors produced by adipogenic marrow cells that negatively influence bone healing in response to age and high-fat diet feeding.^127^ Nine-day treatment with sitagliptin increased the shift toward osteogenic progenitors in the tibia, suggestive of enzymatic activity regulating differentiation.^127^

## DPP4 Expression in Mature Adipocytes and Adipocyte Progenitors

Increased DPP4 expression and activity have been consistently associated with obesity (increased body mass index and excess adipose tissue in humans).^26,35,96,122,128^ In addition, leptin concentrations and the size of adipocytes in both visceral and subcutaneous depots positively associate with DPP4, whereas adiponectin levels negatively correlate with circulating DPP4.^128^ Given its strong correlation with increased adipose tissue accumulation, the origin of obesity-induced circulating DPP4 was originally proposed to be the mature adipocyte.^122,128,129^ Consistent with this idea, the genetic elimination of adipose tissue DPP4 triggers beneficial adipose tissue remodeling and improved hepatic insulin sensitivity in diet-induced obesity.^130^ In addition, in 1-year-old obese mice fed a high-fat, high-cholesterol diet (HFHC), it was determined that a small portion of circulating DPP4 originates from adiponectin + cells.^26^

Recent single-cell RNA sequencing combined with elegant microscopy evidence suggests that DPP4 expression is limited in adipose tissue to multipotent progenitors, which exist within the reticular interstitium and give rise to intercellular adhesion molecule 1 (ICAM)+ and CD142+ preadipocytes.^131^ Previous studies are consistent with *Dpp4* expression on progenitor populations as decreased DPP4 expression is associated with early steps in adipocyte differentiation, including adipocyte maturation.^132^ However, it is possible that in states of obesity DPP4+, mesenchymal progenitor cells are depleted, which reduces the population of preadipocytes disrupting the required adipose tissue hyperplasia, shifting to maladaptive hypertrophy within visceral depots.^131^ This concept requires further investigation.

## DPP4 Within the Adipose Tissue Stromal Vascular Fraction

Dipeptidyl peptidase-4 expression in adipose tissue is much more prevalent in the stromal vascular fraction than in the adipocyte fraction, particularly under conditions of high-fat feeding^26^ Increased DPP4 expression in obese humans and *ob/ob* mice is observed in populations of dendritic cells and macrophages isolated from visceral adipose tissue.^22^ However, the increase in circulating DPP4 observed with increased adipose tissue accumulation is entirely unaffected in mice lacking DPP4 in CD45+ immune cell populations,^30^ suggesting that the majority of dysregulated circulating DPP4 due to obesity was not originating from CD45+ immune cells.

## DPP4 as a Biomarker of Metabolic Liver Disease

The liver has been proposed as a primary source of circulating DPP4.^133^ Within the liver, DPP4 is expressed in both hepatocytes and nonparenchymal cells, yet obesity only increases *Dpp4* expression in hepatocytes.^134^ Analysis by Baumeier et al^135^ calculated by total weight predicted that in mice, the majority of DPP4 protein release came from the liver. Several groups have identified that circulating DPP4 levels are also positively associated with NAFLD.^20,136^ In humans, plasma DPP4 expression and activity are increased in patients with NAFLD and have a strong correlation with liver fat content.^135^ Higher hepatic *DPP4* expression and elevated plasma levels are observed in patients with NAFLD compared with healthy subjects.^20^ Nonbiased, discovery-based proteome profiling in the plasma from patients with T2D, NAFLD, and cirrhosis demonstrated that DPP4 protein levels significantly correlated with concentrations of liver enzymes used currently as markers of liver damage, including alanine aminotransferase (ALT), aspartate transaminase (AST), alkaline phosphatase, and gamma-glutamyl transferase.^137^ Indeed, methylation at CpG sites in the *Dpp4* southern shore has been reported to regulate the expression of *Dpp4* in the liver,^138^ but not in adipose,^135^ kidneys, or brain^138^ of mice, suggesting liver-specific transcriptional regulation in disease states.

## DPP4 as a Hepatokine

Consistent with the role of hepatocyte-derived DPP4 in metabolic disease, Ghorpade et al^35^ identified DPP4 as a circulating casual factor downstream of hepatic CAMKII signaling, which promoted macrophage chemoattractant protein (*Mcp1*), interleukin 6 (*Il6), Tnfa*, and *il-1b* expression and crown-like structures within visceral, but not inguinal or brown adipose tissue. Varin et al^26^ a confirmed these findings in *Dpp4*^*Hep−/−*^ mice, which confirmed that the 40% increase in circulating DPP4 under high-fat diet feeding or obesity is hepatocyte-derived. Similarly, in the absence of hepatic *Dpp4*, reduced levels of F4/80 (*Adgre1), Il2*, C-C chemokine ligand 2 (*Ccl2)*, and *Tnfa* were observed in both the livers and gonadal adipose tissue of HFHC-fed *Dpp4*^Hep−/−^mice.^26^ In both models, reductions in hepatic DPP4 resulted in improvements in whole-body insulin sensitivity, and the effects could not be replicated by treatment with a DPP4i.^26,35^ The HFHC-fed mice treated with the DPP4i MK-0626 demonstrated no changes in cytokine expression in the liver, epididymal fat, or plasma. Also, patients with T2D treated for 4 weeks with the DPP4i sitagliptin failed to demonstrate any significant changes in circulating concentrations of inflammatory cytokines, chemokines, or growth factors.^139^ Conversely, the gonadal white adipose tissue isolated from mice with a hepatocyte-specific overexpression of *Dpp4* (1.6-fold increase in hepatic protein and a 2-fold increase in circulating) demonstrated increased markers of macrophage infiltration (F4/80) and inflammatory cytokines (TNF-α and MCP1) and increased leptin:adiponectin ratios.^135^ Consistent with a direct effect on insulin sensitivity, insulin treatment of HepG2 hepatoma cells and primary mouse hepatocytes infected with adenovirus to overexpress DPP4 demonstrated a reduction in insulin-stimulated Akt phosphorylation.^135^ Incubation of adipocytes, skeletal muscle cells, and smooth muscle cells with DPP4 reduced Akt phosphorylation induced by insulin in a dose-responsive manner, suggesting these signaling effects are direct.^122^

Hypoxia-inducible factor-1 α (*Hif1a)* is also elevated in obesity, and hepatocyte-specific elimination of *Hif1a*, but not *Hif2a*, decreased the upregulation of hepatic *Dpp4* observed with an excess of adipose tissue.^96^ These data suggest that hypoxia may be an initiating factor for the shedding of DPP4 and are consistent with the model proposed by Chowdhury et al.^140^ Deletion of hepatocyte *Hif1a*, which prevents obesity-induced increases in hepatic DPP4, demonstrated increased active portal GLP-1 and improved glucose tolerance.^96^ The lack of upregulation of *Dpp4* within the liver in *Hif1a*^−/−^ mice was associated with a decrease in phosphorylated p65 NF-κB levels,^96^ confirming that elimination of the obesity-induced increase in hepatic DPP4 is linked to reduced inflammation. It has been difficult to dissect the temporal relationship between lipid accumulation, inflammation, and insulin resistance, as mice with hepatic overexpression of DPP4 exhibited increased liver TG accumulation and adipose tissue accumulation beginning at 20 weeks and persisting throughout the 30-week study. They also exhibited increases in fatty acid transporter CD36.^135^ Elimination of *Dpp4* from hepatocytes with siRNA from mice with diet-induced obesity or *ob/ob* mice reduced circulating nonesterified fatty acids (NEFA), effects not replicated with sitagliptin treatment. However, both lipogenesis and oleic acid uptake were similar in both control and DPP4 overexpressing hepatocytes,^135^ and genetic elimination of *Dpp4* specifically from hepatocytes did not lead to any changes in NEFA, TG, or liver lipid accumulation.^26^ These data suggest aberrant communication between adipose tissue and the liver can be the main driver of DPP4’s role in insulin resistance and inflammation. The main cell type identified to respond to liver-derived DPP4 is the adipose tissue macrophage (ATM). In vitro studies have also identified protease-activated receptor 2 (PAR2) as a potential target of soluble DPP4 action in smooth muscle cells^141^ and in cultured human coronary artery endothelial cells.^129^ Ghorpade et al^35^ demonstrated that both PAR2 and caveolin-1 signaling were instrumental in stimulating inflammation in ATM by using an intraperitoneal injection of siRNA encapsulated in micrometer-sized glucan shells to specifically target signaling in ATMs. In addition, they propose that activated factor X may signal synergistically with DPP4 and activate extracellular signal-regulated kinase (ERK1/2) and NF-κB to increase downstream expression mediators of inflammation, including MCP-1, IL-6, and TNF-α.^35^ Indeed, disrupting protein-protein interactions with DPP4, but not DPP4 inhibitor treatment, reduced adipose tissue inflammation,^22^ consistent with a noncatalytic mechanism of action.

## DPP4 Inhibitors and Metabolic Liver Disease

Both diet-induced obese mice and *ob/ob* mice treated with the DPP4i linagliptin improved glycemic parameters and reduced the liver fat content and markers of inflammation, suggesting potentiation of DPP4 substrates may have short-term benefit on liver metabolism.^142^ More recently, Kawakubo et al^143^ treated a genetically obese melanocortin 4 receptor-deficient mouse (model of insulin resistance, hepatic steatosis, nonalcohol steatosis hepatitis [NASH], and hepatocellular carcinoma) with a DPP4i which prevented the progression of simple steatosis to NASH, and decreased hepatic crown-like structures and expression of inflammatory and fibrosis-related genes. Also, in randomized control trials with patients treated with sitagliptin (100 mg) for 1 year, biopsy demonstrated reduced steatosis and ballooning.^144^ In addition, in further studies, sitagliptin (100 mg) decreased ALT and AST and improved histological scoring.^145^ However, adding to the complexity of interpretation, randomized controlled trials of patients with liver disease where patients treated with alogliptin (25 mg/day for 12 months),^146^ vildagliptin (50 mg, twice a day for 6 months),^146^ or sitagliptin (100 mg for 12 months)^147^ reports confirm failure to provide any clear benefit.

## DPP4 and Fibrosis

Shigeta et al discovered that the activity of the membrane-bound form of DPP4 is elevated in a diabetic rat model, but reduced in their normoglycemic counterparts. As a result, circulating stromal-derived factor 1 (SDF-1), angiogenesis, and the number of CXC chemokine receptor–positive/vascular endothelial growth factor receptor–positive (CXCR+KDR+) endothelial progenitor cells were decreased, while there was an increase in fibrosis. The opposite was observed in *Dpp4*^−/−^ and normoglycemic control rats.^148^ CXCL12 signaling has been proposed to promote liver fibrosis by recruiting immune cell populations and has also been linked to the development of hepatocellular carcinoma,^149^ although it is unclear to date whether DPP4i can potentiate this process. Dipeptidyl peptidase-4 has been demonstrated to define fibroblast populations within human skin biopsies,^150^ and DPP4+ fibroblasts have been shown to express higher levels of myofibroblast markers and collagen,^151^ but whether these pathways are relevant in liver disease remains to be determined. Treatment of mice predisposed to fibrosis by treatment with bleomycin or chronic graft versus host disease with DPP4i has resulted in reduced fibrosis and inflammation, and DPP4i have also been found to promote the migration of keratinocytes, and reduce collagen synthesis and deposition, limiting scar formation.^152^

## Future Directions

The substantial body of preclinical evidence in genetic mouse models linking cell-specific actions of DPP4 with insulin resistance, obesity, and NAFLD requires further confirmation and mechanistic studies in patient populations and human model systems to confirm its role as a biomarker or causal agent in disease progression. The novel discovery of the fate of several DPP4+ progenitor cell populations illustrates additional metabolic pathways that may also contribute to the regulation of glucose, insulin sensitivity, and metabolic disease. These observations solidify the need for further elucidation of how DPP4 regulates these pathways—through catalytic activity and cleavage of substrate peptides or direct protein-protein interactions.
